# Exploring Silicone-Induced Lupus Through a Case Report

**DOI:** 10.31138/mjr.171224.lau

**Published:** 2025-07-11

**Authors:** Georgios A. Drosos, Paraskevi V. Voulgari, Alexandros A. Drosos

**Affiliations:** Rheumatology Clinic, Department of Internal Medicine, Medical School, University of Ioannina, Ioannina, Greece

**Keywords:** ASIA syndrome, silicone implants, lupus, autoantibodies, autoimmunity

## Abstract

Silicone is a chemical compound that is composed of one silicone atom and two atoms of oxygen. It has a variety of clinical applications such as breast and joint implants, intraocular lenses and others. Silicone is associated with a variety of autoimmune/inflammatory syndromes induced adjuvant, called ASIA syndrome, among them is lupus development. A 48year-old woman who had silicone breast implantation bilaterally 4 months earlier, presented to us with abrupt, extensive erythematosus skin manifestations affecting her face, nose and lips. She presented annular lesions affecting the upper back and erythematosus lesions affecting the palms of both hands. She manifested also epidermal necrolysis involving the breasts, areolas and nipples bilaterally. Laboratory evaluation revealed low white blood cells, positive antinuclear antibodies at a titre of 1/320, fine speckled pattern and Ro(SSA) and Smith(Sm) antibodies. The patient satisfied the classification criteria for lupus and the proposed criteria for silicone induced ASIA syndrome. She was treated with prednisone 40mg/day plus hydroxychloroquine 400mg/day with excellent results. Thus, through the above case we review and discuss the relevant literature of silicone induced lupus. This is a unique described case with such extensive and severe skin manifestations induced from silicone. To this end, physicians must be aware and recognise these symptoms and signs of patients exposed to silicone and treat them promptly and appropriately.

## INTRODUCTION

Silica or silicone oxide is a chemical compound that is composed of one silicone atom and two atoms of oxygen (SiO2). Naturally, it is found as sand or quartz.^3^ Silicones have a variety of clinical applications such as breast and joint implants, testicular prostheses, intraocular lenses and others.^1,2^ The main purpose of implantation is for cosmetic reasons, although a minority of silicone breast implants (SBIs) are used as part of breast reconstruction in women with mastectomy due to breast cancer. Initially, silicone was considered as a biologically inert material, however, through the decades many autoimmune rheumatic diseases (ARD) and inflammatory phenomena have emerged among them scleroderma, Sjögren’s syndrome (SS), rheumatoid arthritis (RA), vasculitis, and systemic lupus erythematosus (SLE),^3^ as the case we present below. All the above belong to the autoimmune/inflammatory syndrome induced by adjuvant (ASIA).^4,5^ Thus, through this case we review the clinical, immunological, pathophysiological and therapeutic finding of silicone induced lupus. To this end, we have searched through Pub-Med and Scopus using the following words: “silicone” AND “lupus”, “silicone” AND “skin reactions”, “silicone” AND “autoantibodies”, “silicone” AND “widespread skin reactions”, “silicone” AND “extensive skin reactions”, “silicone” AND “autoimmune phenomena”.

## CASE PRESENTATION

**Figure 1.**
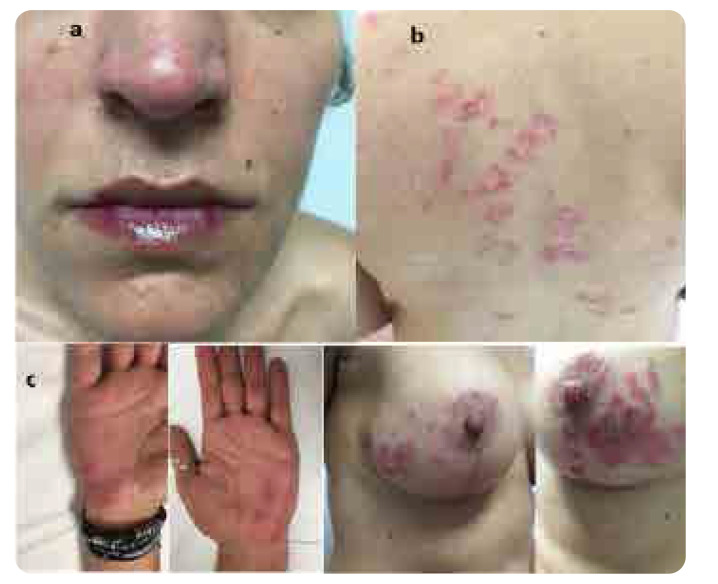
(**a**) Erythematosus skin lesions affecting the face, nose and lips in a photosensitivity distribution. (**b**) Annular skin lesions affecting the upper back are shown. (**c**) Erythematosus skin rashes affecting the palms of the hands are noted bilaterally. (**d**) Erythematosus epidermal necrolysis affecting both breasts, areolas, and nipples are evident.

A 48-year-old housewife visited us complaining about the presence of acute skin reactions, affecting many parts of her body, manifested four weeks earlier. Past medical and family history were unremarkable. She denied photosensitivity, Raynaud’s phenomenon, mucosal ulcers, hair loss and arthritis. She was no smoker and received no medications nor other substances. Clinical examination revealed a woman in acute distress with erythematosus skin lesions affecting her face, nose and lips in a photosensitivity distribution (**Figure 1a**). She presented annular skin lesions affecting the upper back (**Figure 1b**). In addition, erythematosus skin rashes affecting the palms of the hands were noted bilaterally (**Figure 1c**), as well as erythematosus epidermal necrolysis affecting both breasts, areolas and nipples were evident (**Figure 1d**). During the clinical inspection and palpation of the breasts we noticed incision scars under both breasts. Asking her about these signs, she answered that she had undergone implantation of both breasts, four months earlier, for cosmetic reasons. The rest of physical examination was negative. Laboratory investigation revealed no anaemia (haemoglobin 12.9gr/dl), neither thrombocytopenia (platelets 210x10^3^/μL). However, she had low white blood cells (WBC) 3.5x10^3^/μL. C-reactive protein was normal, while the erythrocyte sedimentation rate (ESR) was 51mm/h. Urine analysis was normal, and the rest of laboratory tests were within normal limits. On the other hand, anti-nuclear antibodies (ANA) was positive in a titre of 1/320, in a fine speckled pattern. The double strand (ds) DNA was negative, while Ro(SSA) and Smith(Sm) antibodies were both positive. The complement levels (C3, C4) and anti-cardiolipin antibodies were within normal limits. The patient satisfied the 2019 classification criteria for SLE ^6^ and the proposed diagnostic criteria for ASIA syndrome.^7^ Thus, the diagnosis was silicone-induced lupus.

**Figure 2.**
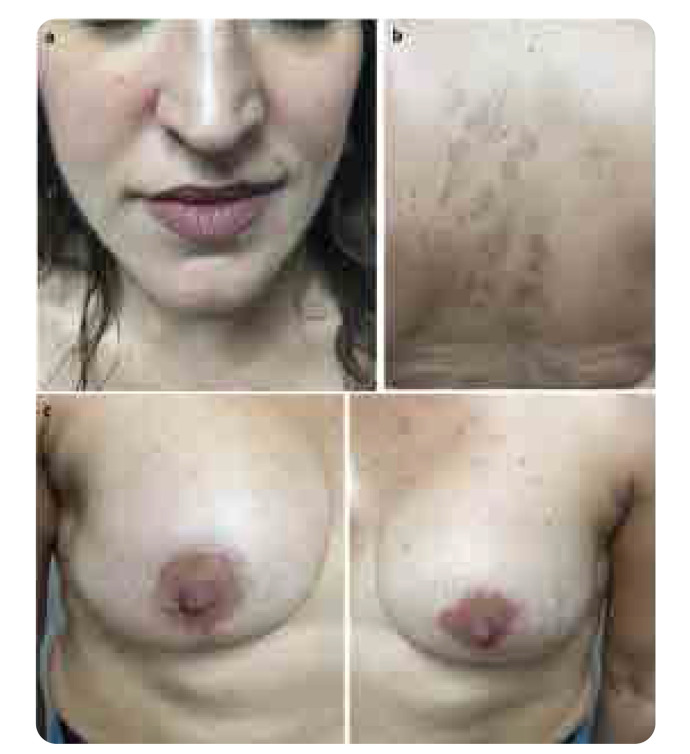
Six months after treatment, the skin manifestations had almost settled down (**a, b, c**).

She was treated with prednisone 40mg/day and hydroxychloroquine (HCQ) 400mg/daily. Two months later she had an excellent clinical and laboratory response, with normalisation of WBC and ESR. Prednisone was tapered. Six months later the skin manifestations had completely settled down, prednisone was stopped, and she continued receiving the same dose of HCQ (**Figure 2**). On an annual re-evaluation she had no skin lesions, with normal laboratory tests and HCQ was tapered to 200mg/daily. The patient had no explantation of the silicone implants.

## EPIDEMIOLOGY

Several epidemiological studies have investigated the association between SBIs and ARD development, reporting inconsistent results. Indeed, in the past few decades, there has been a growing body of evidence (case reports, case series, and case studies) linking SBIs with many ARD and inflammatory phenomena development.^8–12^ This issue has generated controversy in literature. However, despite the abundance of large epidemiological studies that show the association of SBIs with ARD development, the results remain inconclusive and the debate regarding the safety of SBIs still remains.^9^ SBIs have been linked to ARD and clinical symptoms such as arthralgias, arthritis, muscle pain, fatigue, weakness, Raynaud’s phenomenon, erythematosus skin rashes, dry eyes and the presence of autoantibodies, mostly ANA, dsDNA, and others.^3,4,8,10^ In addition, the association between silicone and ARD has also been replicated in animal model studies. In fact, silicone gel, or silicone oil implantation has been showed to increase the dsDNA titre in MRL lpr/lpr mice,^13^ while in the collagen induced arthritis mouse model, increased the susceptibility to arthritis.^14^ Furthermore, silicone-gel injection induced proteinuria and autoimmune haemolytic anaemia in NZB mice.^12^ There are no epidemiological studies to investigate the association of SBIs, with a single or separate disease for example lupus, RA or SS development, but the association included the whole spectrum of ASIA syndrome. Hennekens et al., in a large retrospective study among 10830 healthy female professionals with SBIs, found a relative risk (RR) of 1.24 (95% CI 1.08–1.41), for any self-reported ARD in comparison to those of SBIs-free healthy controls.^15^ In a systematic review Balk et al. found an increased risk of SS and RA in women with SBIs. ^9^ In a large cross-sectional study by Watad et al. there was an association between SBIs and the presence of ARD. SS, scleroderma, and sarcoidosis were the disorders mostly associated with SBIs, while SLE was not so frequently found.^16^

## PATHOPHYSIOLOGY

Many decades ago, silicone was considered as an innocuous chemical element. Today, this notion has been refuted. Silicone has several effects in the immune system, causing dysregulation of the innate and adaptive immune response. Silicone gel can migrate outside the outer shell after SBI rapture and migration occurs throughout the body, the so called “gel bleed”.^17–19^ Thus, it induces local and systemic reactions. Silicone acts as a foreign body and inflammatory responses to silicone, such as granulomatous skin reactions and lymphadenopathy proximal to silicone implants have been observed. Furthermore, small particles of silicone have been observed far from the original site, suggesting that some particles of silicone detaches and migrates through the lymphatic and systemic circulation to other organs. They act then as adjuvants and activate the immune system and start a systemic inflammation and stimulate the generation of antibodies against silicone and against various components of the body. Indeed, the presence of antibodies against silicone and autoantibodies such as ANA, dsDNA and others have been demonstrated in some patients with SBIs. Although, antibodies against silicone were detected in many studies, no association with clinical manifestations were observed. More specifically, silicone triggers a local foreign body reaction characterized by infiltration of inflammatory cells such as macrophages, giant cells and T cells. Silicone particles are then captured by macrophages, activate the NALPS inflammasome and release interleukin (IL)-1β, causing systemic inflammation, activation of T and B cells with the production of many autoantibodies.^20–22^ The mechanisms which have been proposed are mostly the theory of molecular mimicry and the polyclonal activation of B cells. Indeed, silicone as an adjuvant acts by mimicking specific sets of evolutionarily conserved molecules which include: liposomes, lipopolysaccharides, collagen, fibronectin, endocytosed nucleic acids such as dsDNA, ssDNA, nucleosome, and others. Other mechanisms of silicone induced autoimmunity are the polyclonal activation of B-cell cells, the bystanders activation which enhances cytokine production, with expansion of auto-reactive T-cells and epitope spreading which accelerates the local activation of macrophages and dendritic cells and the over processing of antigens.^20– 22^ Thus, SBIs can cause chronic inflammation which lead to polyclonal activation of B-cells with generation of autoimmunity, or can switch to monoclonality leading to lymphoma development.^22,23^

## CLINICAL MANIFESTATIONS

The clinical manifestations of ASIA syndrome are several, manifested mostly as vague systemic symptoms affecting many organs and systems, or as clinical entities, especially ARD, or as an inflammatory disorder. The main symptoms and clinical manifestations include: fatigue, myalgias, arthralgias, low grade fever, weakness, memory loss, depression, and generalised pain. The most common ARD development are: RA, SS, scleroderma, lupus, sarcoidosis, Still’s disease, spondylarthritis, psoriasis, anti-phospholipid syndrome.^24–28^ The diagnosis of ASIA syndrome requires two major, or one major and two minor criteria, as proposed by Shoenfeld Y and Agmon-Levin N. ^7^ The major criteria include: 1.Exposure to external stimuli, before the onset of symptoms, 2.The appearance of typical clinical manifestations a) myalgia, myositis or muscle weakness b)arthralgias and/or arthritis, c)chronic fatigue, up-refreshing sleep, or sleep disturbances, d)Neurological manifestation (demyelination), e)cognitive impairment, memory loss, f)fever, 3.Typical histological findings after biopsy of affected organs, 4. Removal of offending agent results in improvement of symptomatology. The minor criteria comprise: 1. Appearance of antibodies directed against the adjuvant suspected to be involved, 2. Secondary clinical manifestations (irritable bowel syndrome, interstitial cystitis etc), 3. Evolvement of an autoimmune disease (scleroderma, multiple sclerosis etc), 4. Antigens specific for human leucocytes (HLA DRB_1_, HLA DQB_1_).^7^

## MANAGEMENT

Consensus statements focused on the management of ASIA syndrome are lacking. Removing or replacing the silicone or the adjuvant showed a decrease of ASIA symptoms and clinical manifestations of about 60–75%. Removal of silicone is the most effective treatment. However, in patients with manifestations of ARD, this approach might not be effective or sufficient.^29–31^ Thus, additional treatment is required, depending on the clinical manifestations of the disease development. The treatment comprises the use of medium to high doses of corticosteroids (CS), the use of conventional synthetic disease modifying antirheumatic drugs (csDMARDs), such as methotrexate (MTX), leflunomide (LFN), HCQ and immunosuppressive drugs such as cyclophosphamide (CP), azathioprine (AZA), rituximab and also pulse CS therapy.^9–11^ The majority of these patients responded well to the above treatment choice, as the patient we presented above (**Figure 1** and **Figure 2**).However, removal of silicone or adjuvant is the most effective treatment.^29–31^

## DISCUSSION

SBIs are associated, in a proportion of patients, with symptoms and ARD manifestations of ASIA syndrome. Despite the changes in the principal constitutes of the silicone implants during the past 30 years, silicone remains an adjuvant that elicits inflammatory phenomena. Indeed, in a study by Colaris et al. 100 patients who were diagnosed in 2014 were compared with 100 historical patients with adjuvant breast implants diagnosed between 1985–1992. It was found that silicone related diseases have not changed during the last 3 decades.^3^ The diagnosis of ASIA syndrome and ARD requires the identification of temporal association between SBIs and the development of clinical manifestations, for example lupus, in women without pre-existing lupus or another ARD, by applying the proposed diagnostic criteria.^7^ The clinical manifestations of lupus development after SBIs are expressed mostly with erythematosus skin lesions affecting the face, in a butterfly distribution, and other parts of the body with annular, psoriasiform or discoid lesions in a photosensitivity distribution, as the patient we presented above (**Figure 1**). Other clinical manifestations include arthralgias, arthritis, synovitis, pleurisy, constitutional symptoms.^32–34^ However, kidney and central nervous disease are uncommon. The occurrence of ASIA symptoms development after SBIs, varies among studies ranging between 3 to 36 months. The meantime between implantation and the onset of clinical manifestations is about 2 years.^15–24^ The majority of women received SBIs for cosmetic reasons (78%), while the rest (22%) received SBIs for reconstruction following mastectomy after breast cancer management, or because of a genetic predisposition for breast cancer gene mutation (BRCA).^15–24,32^ The most common symptoms after SBIs, are fatigue, myalgias, arthralgias, low grade fever, dry eyes and ARD development such as RA, SS, scleroderma, and sarcoidosis, while the development of lupus is less frequently found.^15,16^ As regards the presence of antibodies against adjuvant and the autoantibodies detected, their occurrence varies between studies. ANA was reported in about 23%, dsDNA in 11%, rheumatoid factor 9%. However, antibodies are often nonspecific for ARD. Their presence may contribute to a diagnosis; however, their absence does not exclude its diagnosis. Silicone may cause a local inflammatory response, but silicone particles can migrate through the body and can be detected far away from the breast, causing a chronic inflammatory response of the immune system.^24–28,32^ Indeed, our patient had severe erythematosus epidermal necrolysis affecting both breasts. This could be a local inflammatory response to silicone, however several widespread cutaneous lesions have been observed affecting many parts of her body, suggesting a more systemic inflammatory response. It is postulated that the polyclonal activation of B-cells may result in monoclonality and the lymphoma development, especially anaplastic large cell lymphoma (ALCL).^24,30^ As regards the management of ASIA syndrome, explantation is the most effective treatment and should be advised to patients with complains of ASIA syndrome. Indeed, de Boer et al. showed that explantation of the silicone breast improved the symptoms of about 75% of the patients.^30^ Explantation did not influence the presence of autoantibodies.^29,30,32^ It is reported that several women still suffer from ASIA symptoms after explantation of silicone, possibly because silicone particles are present throughout the body, causing continuous chronic inflammation. In these cases, the use of CS, along with csDMARDs are more effective.^5,9,16,24,32^ In our case, the implants were not removed because she responded very well to the treatment and refused any further intervention.

Our patient had an SBI in both breasts 4 months before the clinical symptoms developed. She satisfied the proposed criteria for SLE related to SBIs.^7^ The early treatment with medium to high doses of CS along with the use of HCQ resulted in an excellent clinical response (**Figure 2**). We also considered other possibilities such as the coincidence of a subclinical pre-existing lupus and SBI procedure or the presence of a subclinical lupus and its flaring after SBI surgery. These possibilities are difficult to be excluded. However, our patient had no history of photosensitivity, skin rashes, oral ulcer, arthritis, or other signs and symptoms or pre-existing blood or immune abnormalities suggesting lupus before SBIs. Predisposing factors for ARD development in patients with silicone implants are very difficult to demonstrate. Despite the changes in the principal constitutes of the silicone implants during the last 30 years, it seems that silicone remains an adjuvant that elicits inflammation and autoimmune phenomena.^3^ Nevertheless, some recommendations were made to avoid silicone implants: individuals previously diagnosed with an ARD, or genetic predisposition for hyperactive immune system, should not be considered as candidates for silicone implantation.^35^

## CONCLUSIONS

Silicone breast implants have been used for many decades for cosmetic surgery and reconstruction. Silicones are polymeric compounds sharing a silicon-oxygen chain with varying organic side groups, that can activate innate and adaptive immune system with the development of many autoimmune diseases, among them lupus. We described a unique case of acute cutaneous lupus development, with extensive and severe skin manifestations induced by silicone. Thus, physicians should not forget that SBI is not an innocent procedure. A minute and careful past medical history, clinical examination and early recognition with prompt therapeutic interventions are mandatory.

## STATEMENT OF ETHICS AND CONSENT

This study was performed in accordance with the Helsinki Declaration of 1964 and its later amendments. All presented material is published after written consent of the patient, although sensitive data and personal details are not included in the publication. The approval of the photos to be published, was on the last appointment on September 3, 2024.

## AUTHOR CONTRIBUTIONS

All authors contributed to the final manuscript. G.A Drosos: Literature screening, manuscript preparation, P.V. Voulgari: Review and editing, A.A. Drosos: Conceptualisation, final review. All authors have read and approved the final submitted version.

## CONFLICT OF INTEREST

The authors declare no conflicts of interest.

## FUNDING

None.
